# Draft genomes of multidrug-resistant *Acinetobacter baumannii* isolates from an agriculturally-dominated watershed and dairy cattle feces

**DOI:** 10.1128/mra.00829-25

**Published:** 2025-10-17

**Authors:** Cecilio Valadez-Cano, Izhar U. H. Khan, Jiacheng Chuan, Xiang (Sean) Li, David R. Lapen, Suzanne Gerdis, Jeremy Dettman, Wen Chen

**Affiliations:** 1 Ottawa Research & Development Centre, Science & Technology Branch, Agriculture and Agri-Food Canada 6337, Ottawa, Ontario, Canada; 2 Canadian Food Inspection Agency, Charlottetown Lab, Charlottetown, Prince Edward Island, Canada; University of Wisconsin-Madison, Madison, Wisconsin, USA

**Keywords:** *Acinetobacter baumannii*, antibiotic resistance, carbapenem resistance

## Abstract

*Acinetobacter baumannii* is an opportunistic human pathogen that primarily affects individuals with compromised immune systems. Here, we report the draft genomes of six *A. baumannii* strains isolated from animal fecal sources and mixed-use but primarily agricultural surface waters. These genomes contain a diverse array of antibiotic resistance and virulence-associated genes.

## ANNOUNCEMENT

*Acinetobacter baumannii* is a major opportunistic pathogen associated with severe (co)infections in immunocompromised individuals. The ability to acquire and express diverse antimicrobial resistance (AMR) determinants has driven the rise of multidrug-resistant and extensively drug-resistant strains (1–3). Its resilience in diverse environments supports persistence and facilitates dissemination into healthcare settings (4, 5). Particularly concerning are carbapenem-resistant (CRAB) strains, which the World Health Organization designates as ‘critical’ priority pathogens due to limited treatment options and high clinical burden (6).

Strains SNC-24, SNC-253, SNC-5, and SNC-9 were isolated from surface waters in an agriculturally dominated but mixed-use watershed, whereas CF0141 and CF0140 were recovered from dairy cattle feces in the South Nation River basin (45.170, -75.079), eastern Ontario, Canada. Collected samples were suspended in 1× phosphate-buffered saline and serially diluted 10-fold. Bacteria were cultured on Karmali agar (Thermo Fisher Scientific, KS, USA) supplemented with amphotericin B, cefoperazone, sodium pyruvate, and vancomycin, and plates incubated at 42°C under microaerophilic conditions (5% O_2_, 85% N_2_, and 10% CO_2_) for 48 h. Single colonies were subcultured to ensure purity and stored at −20°C for further analysis. Genomic DNA was extracted with the DNeasy UltraClean microbial kit (Qiagen, MD, USA). Identification was performed by 16S rRNA sequencing with primers pA-F (5’ – AGA GTT TGA TCC TGG CTC AG- 3’) and pH-R (5’ – AAG GAG GTG ATC CAG CCG C - 3’). Sequencing was performed on an ABI PRISM 3130XL Genetic Analyzer (Applied Biosystems) and BLAST analysis confirmed 100% identity to *A. baumannii* 16S rRNA.

Whole-genome sequencing was performed using the Illumina NextSeq 500 platform (2×150 bp) using the Nextera DNA Flex Library Prep kit following manufacturer’s instructions. Reads were trimmed with *Atria v3.1.2* (first 10 bp removed) (7), quality-checked with *FastQC v0.12.1* (8) and *MultiQC v1.19* (9), and assembled *de novo* with *MEGAHIT v1.2.9* (10), discarding contigs <500 bp. Assembly quality was evaluated with *CheckM v1.2.2* (11), and taxonomy confirmed with *GTDB-Tk v2.3.2* (12). Scaffolding was performed using *RagTag v2.1.0* (13) with *A. baumannii* K09-14 (GCA_008632635.1) as reference. Gene annotation was conducted with *Prokka v1.14.6* (14). Virulence factors were identified via *BLASTp v2.12.0* (15) against the *Virulence Factor Database* (16), and AMR genes were detected using *AMRFinderPlus v3.11* (17). Default parameters were applied unless stated otherwise.

**Fig 1:**
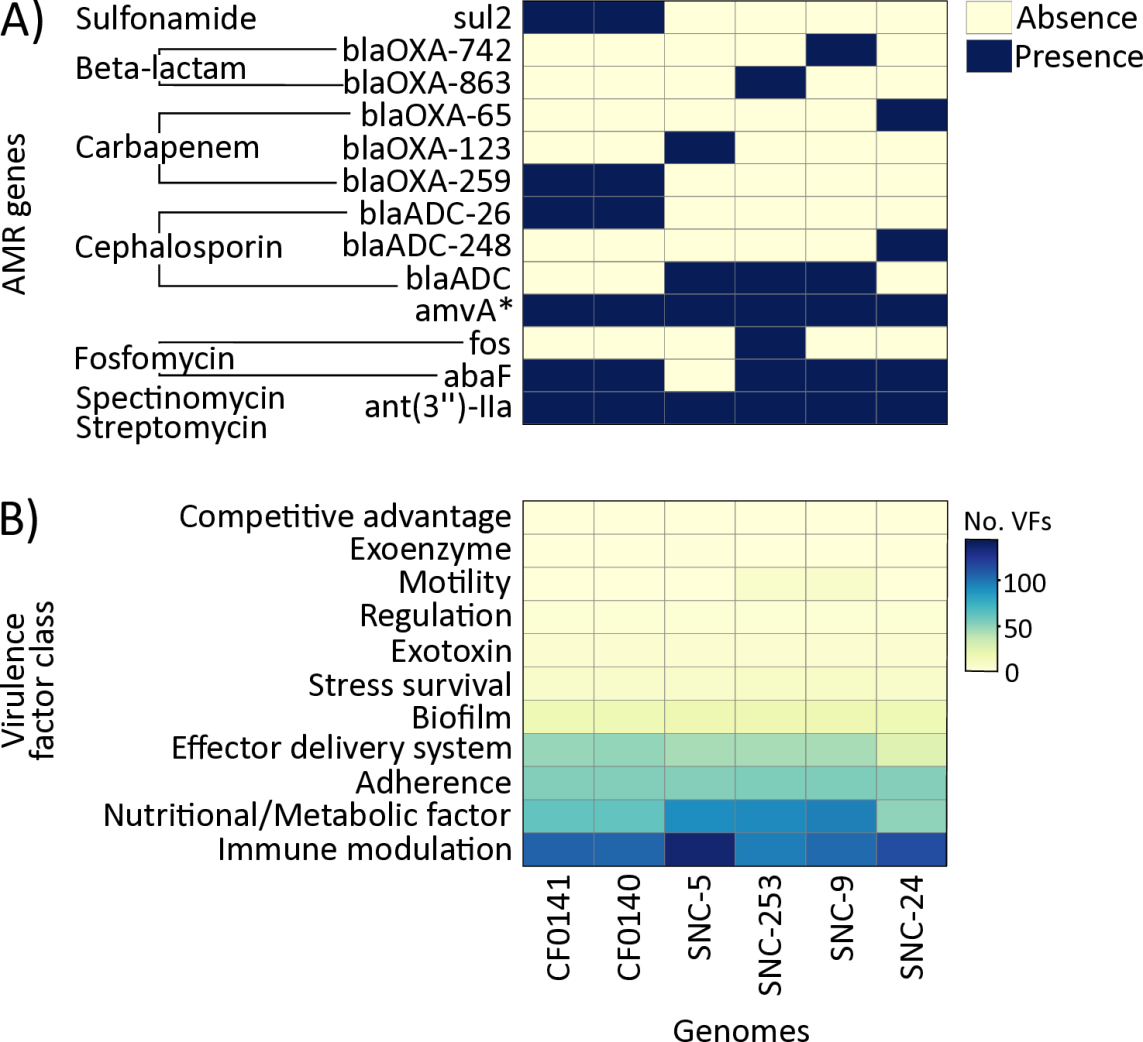
Genome-based assessment of antibiotic resistance and virulence factors in *A*. *baumannii* strains. (**A**) AMR genes identified in six *A. baumannii* genomes and their associated antibiotic resistance profiles. *amvA confers resistance to disinfecting agents, antiseptics, and macrolide antibiotics. (**B**) Number of virulence factor genes categorized by functional class.

High-quality draft genomes (3.71–3.98 Mbp; N50: 3.68–3.92 Mbp) were recovered from all isolates, each with >98% completeness, <1.3% contamination, and 72–140× coverage (Table 1). Isolates were predicted to be resistant to spectinomycin/streptomycin and cephalosporins, with variable resistance to other antibiotics (Fig. 1A). SNC-5, SNC-24, CF0141, and CF0140 were identified as potential CRAB strains, based on the presence of carbapenem resistance genes (Fig. 1A). Virulence factors were predominantly associated with immune modulation (VFC0258), nutritional/metabolic factors (VFC0272), adherence (VFC0001), effector delivery systems (VFC0086), and biofilm formation (VFC0271) (Fig. 1B).

**TABLE 1:** Accession numbers, sequencing and assembly statistics, and genomic features of the six *A. baumannii* strains reported in this study

	*A. baumannii* strains					
	SNC-24	SNC-253	SNC-5	SNC-9	CF0141	CF0140
WGS Accession	JBPSJR000000000	JBPSJQ000000000	JBPSJP000000000	JBPSJO000000000	JBPSJN000000000	JBPSJM000000000
SRA Accession	SRR33813084	SRR33813083	SRR33813082	SRR33813081	SRR33813080	SRR33813079
Total Read Pairs	1,617,312	1,617,336	1,015,731	2,009,428	1,516,751	1,629,001
Coverage	108.7	109	72.7	139.2	105	108.9
Length (bp)	3,965,885	3,944,394	3,712,131	3,737,889	3,859,206	3,984,304
Number scaffolds	9	9	9	4	7	15
N50 scaffolds (Mbp)	3.92	3.92	3.68	3.73	3.81	3.79
Completeness (%)	99.8	99.8	99.8	99.8	98.2	98.6
Contamination (%)	0.7	1.2	0.5	0.8	0.6	0.7
CDS	3,726	3,655	3,437	3,384	3,609	3,732

## Data Availability

The data generated in this study have been deposited in NCBI under BioProject accession no. PRJNA1271542.

## References

[1] Ayoub Moubareck C, Hammoudi Halat D. 2020. Insights into Acinetobacter baumannii: a review of microbiological, virulence, and resistance traits in a threatening nosocomial pathogen. Antibiotics (Basel) 9:119. doi:10.3390/antibiotics9030119 32178356 PMC7148516

[2] Muntean D, Licker M, Horhat F, Dumitrașcu V, Săndesc D, Bedreag O, Dugăeșescu D, Coșniță DA, Krasta A, Bădițoiu L. 2018. Extensively drug-resistant Acinetobacter baumannii and Proteeae association in a Romanian intensive care unit: risk factors for acquisition. Infect Drug Resist 11:2187–2197. doi:10.2147/IDR.S171288 30519056 PMC6233948

[3] Mirzaei B, Bazgir ZN, Goli HR, Iranpour F, Mohammadi F, Babaei R. 2020. Prevalence of multi-drug resistant (MDR) and extensively drug-resistant (XDR) phenotypes of Pseudomonas aeruginosa and Acinetobacter baumannii isolated in clinical samples from Northeast of Iran. BMC Res Notes 13:380. doi:10.1186/s13104-020-05224-w 32778154 PMC7418330

[4] Clark NM, Zhanel GG, Lynch JP. 2016. Emergence of antimicrobial resistance among Acinetobacter species: a global threat. Curr Opin Crit Care 22:491–499. doi:10.1097/MCC.0000000000000337 27552304

[5] Hamidian M, Maharjan RP, Farrugia DN, Delgado NN, Dinh H, Short FL, Kostoulias X, Peleg AY, Paulsen IT, Cain AK. 2022. Genomic and phenotypic analyses of diverse non-clinical Acinetobacter baumannii strains reveals strain-specific virulence and resistance capacity. Microb Genom 8:000765. doi:10.1099/mgen.0.000765 35166651 PMC8942024

[6] Geneva: World Health Organization. 2024. WHO Bacterial Priority Pathogens List, 2024: bacterial pathogens of public health importance to guide research, development and strategies to prevent and control antimicrobial resistance

[7] Chuan J, Zhou A, Hale LR, He M, Li X. 2021. Atria: an ultra-fast and accurate trimmer for adapter and quality trimming. GigaByte 2021:gigabyte31. doi:10.46471/gigabyte.31 36967729 PMC10038132

[8] Andrews S. 2010. FastQC: a quality control tool for high throughput sequence data

[9] Ewels P, Magnusson M, Lundin S, Käller M. 2016. MultiQC: summarize analysis results for multiple tools and samples in a single report. Bioinformatics 32:3047–3048. doi:10.1093/bioinformatics/btw354 27312411 PMC5039924

[10] Li D, Liu C-M, Luo R, Sadakane K, Lam T-W. 2015. MEGAHIT: an ultra-fast single-node solution for large and complex metagenomics assembly via succinct de Bruijn graph. Bioinformatics 31:1674–1676. doi:10.1093/bioinformatics/btv033 25609793

[11] Parks DH, Imelfort M, Skennerton CT, Hugenholtz P, Tyson GW. 2015. CheckM: assessing the quality of microbial genomes recovered from isolates, single cells, and metagenomes. Genome Res 25:1043–1055. doi:10.1101/gr.186072.114 25977477 PMC4484387

[12] Chaumeil P-A, Mussig AJ, Hugenholtz P, Parks DH. 2022. GTDB-Tk v2: memory friendly classification with the genome taxonomy database. Bioinformatics 38:5315–5316. doi:10.1093/bioinformatics/btac672 36218463 PMC9710552

[13] Alonge M, Lebeigle L, Kirsche M, Jenike K, Ou S, Aganezov S, Wang X, Lippman ZB, Schatz MC, Soyk S. 2022. Automated assembly scaffolding using RagTag elevates a new tomato system for high-throughput genome editing. Genome Biol 23:258. doi:10.1186/s13059-022-02823-7 36522651 PMC9753292

[14] Seemann T. 2014. Prokka: rapid prokaryotic genome annotation. Bioinformatics 30:2068–2069. doi:10.1093/bioinformatics/btu153 24642063

[15] Camacho C, Coulouris G, Avagyan V, Ma N, Papadopoulos J, Bealer K, Madden TL. 2009. BLAST+: architecture and applications. BMC Bioinformatics 10:421. doi:10.1186/1471-2105-10-421 20003500 PMC2803857

[16] Liu B, Zheng D, Zhou S, Chen L, Yang J. 2022. VFDB 2022: a general classification scheme for bacterial virulence factors. Nucleic Acids Res 50:D912–D917. doi:10.1093/nar/gkab1107 34850947 PMC8728188

[17] Feldgarden M, Brover V, Gonzalez-Escalona N, Frye JG, Haendiges J, Haft DH, Hoffmann M, Pettengill JB, Prasad AB, Tillman GE, Tyson GH, Klimke W. 2021. AMRFinderPlus and the reference gene catalog facilitate examination of the genomic links among antimicrobial resistance, stress response, and virulence. Sci Rep 11:12728. doi:10.1038/s41598-021-91456-0 34135355 PMC8208984

